# HIV Care Retention and Cost Analysis in Multi‐Month ART Dispensing: A Randomized Controlled Trial in China

**DOI:** 10.1002/jia2.70151

**Published:** 2026-06-24

**Authors:** Wenqi Shang, Yanyan Zhu, Ying He, Xiaolin Li, Qinghai Hu, Hong Sun, Wenqing Geng, Hong Shang, Haibo Ding

**Affiliations:** ^1^ State Key Laboratory for Diagnosis and Treatment of Infectious Diseases NHC Key Laboratory of AIDS Prevention and Treatment National Clinical Research Center for Laboratory Medicine The First Hospital of China Medical University, China Medical University Shenyang China

**Keywords:** antiretroviral therapy, cost‐effectiveness, differentiated service delivery, People living with HIV, multi‐month dispensing, retention in care

## Abstract

**Introduction:**

Multi‐month dispensing (MMD), as a differentiated service delivery model, can reduce the frequency of clinic visits, waiting time and travel costs for clinically stable people living with HIV. This study aimed to evaluate the impact of 6‐month MMD of antiretroviral therapy (ART) on retention and conduct a cost analysis in China.

**Methods:**

We conducted a randomized, non‐blind, non‐inferiority study from December 2022 to March 2023 at The First Hospital of China Medical University. Eligible participants were randomly assigned to a 3‐month dispensing group (*n* = 789) or a 6‐month dispensing group (*n* = 799) and followed up for 18 months. The proportion of patients continuing ART after 18 months, virological suppression rate (<50 copies/mL) and average treatment cost per patient were evaluated. Cox regression analysis was used to compare treatment retention rates and virological suppression rates between groups, while descriptive statistical analysis was applied to assess cost differences. Cost metrics comprised the average cost per clinic visit and the price of ART medications, among other factors. This trial is registered with ChiCTR2200066438.

**Results:**

A total of 1588 participants were included (median age 40.0 years, IQR 34.0–50.0; 94.8% male), with no significant between‐group differences in demographic and clinical characteristics (all *p*>0.05). In the intention‐to‐treat analysis, treatment retention rates at 18 months were 94.9% (749/789) in the 3‐month dispensing group and 94.2% (753/799) in the 6‐month dispensing group. The risk difference (6‐month minus 3‐month) was −0.7% (95% CI −2.9% to 1.5%); non‐inferiority was demonstrated as the lower bound of the 95% CI (−2.9%) exceeded the pre‐specified margin of −5%. Viral suppression rates (<50 copies/mL) were similarly high in both groups in intent‐to‐treat analysis: 94.9% (3‐month) versus 94.2% (6‐month), with no statistically significant difference. Per‐protocol analysis confirmed these findings (viral suppression 97.59% vs. 97.75%; χ^2^ = 0.0421, *p* = 0.8375). In terms of cost, the 6‐month dispensing group had two fewer annual outpatient visits (3 vs. 5), with total treatment costs reduced by 27.7% (¥931.82 vs. ¥1288.85) and work value loss decreased by 16.7% (¥174.20 vs. ¥209.04 yearly).

**Conclusions:**

Six‐month MMD of ART did not reduce treatment retention; instead, it decreased patients’ clinic visit costs, thereby meeting cost‐effectiveness criteria.

**Clinical Trial Number:**

ChiCTR2200066438.

## 1 Introduction

Antiretroviral therapy (ART) is pivotal for HIV control, reducing viral load, improving quality of life and extending survival [1]. However, lifelong medication demands strain healthcare systems and burden patients with frequent clinic visits—entailing transportation and time costs, particularly for remote populations [2]. Missed appointments often disrupt treatment [3, 4, 5, 6].

The WHO recommends differentiated service delivery (DSD) models for clinically stable people living with HIV (PLHIV) [7], with multi‐month dispensing (MMD) as a core component [8]. MMD plays a crucial role in DSD by simplifying care, reducing unnecessary burdens on patients and health systems, and improving retention rates to address these challenges [9, 10]. MMD extends ART refill intervals, reducing visits, costs and healthcare workload without compromising treatment retention compared to frequent dispensing [11, 12]. Notably, the 6‐month MMD (6MMD) matches traditional care outcomes while reducing costs [13], with a South African study showing it averts 0.8–1 DALYs per year per patient and better controls disease [14].

China faces a substantial and evolving HIV burden, with 1.33 million PLHIV and 474,006 cumulative deaths reported by June 2024 [15]. Epidemiological data indicate that AIDS‐related mortality in China has risen consistently over the past two decades, with 22,137 deaths reported in 2023 alone [16]. Epidemiological data indicate that AIDS‐related mortality in China has risen consistently over the past two decades, with 22,137 deaths reported in 2023 alone [17]. Thus, exploring MMD is critical to enhancing treatment adherence, improving outcomes and optimizing healthcare resource allocation.

In China, stable patients on first‐line ART attend clinic every 3 months for refill and assessment, consistent with the standardized follow‐up schedule established under the National Free Antiretroviral Treatment Program (NFATP) since 2003 [18, 19]. Whether 6‐month dispensing maintains outcomes in this setting remains uncertain.

Thus, exploring MMD is critical to enhancing treatment adherence, improving outcomes and optimizing healthcare resource allocation. This study aims to comprehensively assess the clinical efficacy and cost of 6‐month ART dispensing to inform optimal HIV care strategies in China.

## 2 Methods

### 2.1 Study Design and Participants

We conducted a randomized, open‐label, non‐inferiority trial at the HIV Care Clinic of The First Affiliated Hospital of China Medical University in Shenyang, Liaoning Province, northeastern China (December 2022–March 2023). Clinically stable adults (≥18 years) on first‐line ART were eligible, defined per WHO 2021 guidance as: (i) ART duration ≥6 months with good adherence; (ii) HIV RNA <200 copies/mL within 6 months prior; (iii) no active opportunistic infection or OI treatment within 30 days; (iv) not pregnant/breastfeeding; and (v) no chronic comorbidities requiring concurrent HIV clinic management [20]. Exclusions included clinical instability, pregnancy/breastfeeding, non‐communicable diseases and non‐first‐line ART regimens.

### 2.2 Non‐Inferiority Margin and Sample Size

The non‐inferiority margin was pre‐specified at −5 percentage points for the absolute difference in 18‐month retention rates (6‐month minus 3‐month), consistent with the INTERVAL trial [13, 21, 22]. Sample size calculation (95% expected retention, one‐sided α = 0.025, 90% power) yielded 406 per group; enrolling 1588 provided adequate power [12].

### 2.3 Randomization and Follow‐Up

**FIGURE 1:**
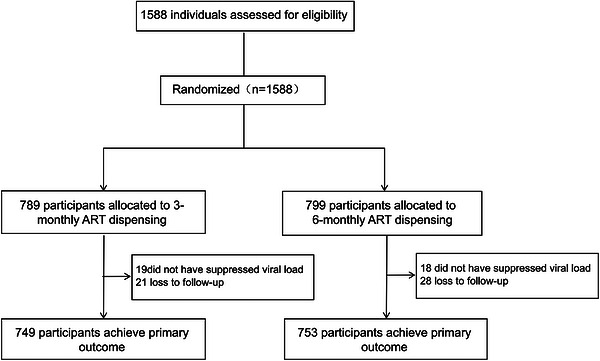
Trial flow diagram. Abbreviation: ART, antiretroviral therapy.

Eligible participants were allocated to 3‐ or 6‐month ART dispensing groups via simple randomization to eliminate selection bias (Figure 1). Consented participants were consecutively numbered; SPSS 27.0 (IBM Corp., Armonk, NY, USA) generated 2500 random numbers matched to enrolment numbers. Sorted random numbers assigned participants to two groups (first/second half). Researchers were blinded to baseline characteristics to avoid subjective bias.

### 2.4 Data Collection

Collected data included demographics (gender, age, marital status, household size, education, employment), clinical indicators (CD4 count, viral load, partner notification rate, ART duration) and healthcare costs (outpatient expenses, lost productivity, travel expenditures).

### 2.5 Outcomes

The primary outcome was retention in care at 18 months (no ≥60 days without ART, consistent with INTERVAL) [13]. Secondary outcomes: viral suppression (<50 copies/mL) and cost. Loss to follow‐up: no visit/refill/contact ≥90 days after last appointment without transfer‐out. Virologic failure: HIV RNA ≥50 copies/mL at scheduled monitoring per WHO [20]; incomplete data classified as failure in intent‐to‐treat (ITT). Both ITT and per‐protocol (PP) analyses were performed.

### 2.6 Cost Analysis

Provider costs (clinic visits, fixed ART care) and patient costs (travel, time, productivity loss) were estimated via micro‐costing. Provider costs include healthcare worker time and utilities allocated per visit, and equipment depreciation allocated per patient‐month. Excluded were ART medication procurement (provided free under national policy), laboratory monitoring (covered by national free policy, identical between groups), building/infrastructure depreciation and administrative/overhead costs (non‐incremental). Productivity loss was calculated using Liaoning Province's average wage standards (published official statistics). Data are presented as mean (95% CI) or median (IQR).​

### 2.7 Statistical Analysis

Data were analysed using SPSS (version 27.0). Continuous variables are presented as mean (SD) or median (IQR), and between‐group comparisons were made using independent samples *t*‐tests or non‐parametric tests (Mann−Whitney U or Kruskal−Wallis), as appropriate. Categorical variables are summarized as counts and percentages, with differences assessed by χ^2^ tests or Fisher's exact test for small sample sizes.

Treatment retention rates across medication dispensing intervals were compared using Cox proportional hazards regression models, with results reported as hazard ratios (HRs) and 95% CIs. For the secondary outcome of treatment failure (viral load ≥50 copies/mL), both ITT and PP analyses were performed. ITT analysis included all randomized participants, with lost‐to‐follow‐up cases considered as treatment failures. PP analysis included only participants who completed the 18‐month follow‐up without major protocol deviations. Pearson's chi‐square test was used to compare treatment failure rates between groups, and Cox proportional hazards regression models were used to assess the risk of treatment failure. A two‐sided *p* value of <0.05 was considered statistically significant. Costs were evaluated using descriptive statistics. A two‐sided *p* value of <0.05 was considered statistically significant.

### 2.8 Ethics and Informed Consent

The study was approved by the Medical Science Research Ethics Committee of The First Affiliated Hospital of China Medical University. All participants provided written informed consent prior to enrolment.

## 3 Results

### 3.1 Clients’ Characteristics of the Study Sample

A total of 1588 participants were included in the final analysis, comprising 789 in the 3‐month medication group and 799 in the 6‐month dispensing group. The majority were MSM (82.2%), with 10.2% reporting heterosexual contact. This reflects China's concentrated epidemic among key populations in urban settings. The median age of participants was 40.0 years (IQR 34.0–50.0), with males accounting for 1505 cases (94.8%). Demographic characteristics, including gender, age, partner disclosure rate, ART duration, marital status, household size, educational attainment and employment status, showed no statistically significant differences between the two HIV‐positive groups (all *p* values >0.05), demonstrating satisfactory baseline comparability (Table 1).

**TABLE 1:** Baseline demographic and clinical characteristics of participants (*n* = 1588).

	Overall participants (*n* = 1588)	3‐monthly dispensing (*n* = 789)	6‐monthly dispensing (*n* = 799)	*p* value
Age	40.0 (34.0−50.0)	39.0 (34.0−50.0)	40.0 (35.0−51.0)	0.3622
Sex				
Male	1505(94.8%)	754(95.6%)	751(94.0%)	0.1597
Female	83(5.2%)	35(4.4%)	48(6.0%)	
HIV transmission route				0.2453
MSM (homosexual + bisexual)	1306 (82.2%)	644 (81.6%)	662 (82.9%)	
Heterosexual contact	162 (10.2%)	77 (9.8%)	85 (10.6%)	
Other/unknown	120 (7.6%)	68 (8.6%)	52 (6.5%)	
Disclosure of HIV status to primary partner				
Yes	1182(74.4%)	601(76.2%)	581(72.7%)	0.8245
No	239(15.1%)	113(14.3%)	126(15.8%)	
Miss or not answered	167(10.5%)	75(9.5%)	92(11.5%)	
Time receiving ART, years	6.6(4.5−8.7)	6.5(4.4−8.5)	6.7(4.7−9.0)	0.0658
Marital status				
Married or long‐term partner	768(48.4%)	372(47.1%)	396(49.6%)	0.2784
Single or no long‐term partner	706(44.5%)	362(45.9%)	344(43.1%)	
Widowed, divorced or separated	114(7.2%)	55(7.0%)	59(7.4%)	
Household size	2(1−3)	2(1−3)	2(1−3)	0.7088
Education (highest level completed)				
No education or less than primary school	23(1.4%)	12(1.5%)	11(1.4%)	0.5686
Primary school	81(5.1%)	40(5.1%)	41(5.1%)	
Secondary school	553(34.8%)	266(33.7%)	287(35.9%)	
University graduate	931(58.6%)	471(59.7%)	460(57.6%)	
Employment status				
Not working	250(15.7%)	127(16.1%)	123(15.4%)	0.3128
Informal employment	452(28.5%)	232(29.4%)	220(27.5%)	
Formal employment	886(55.8%)	430(54.5%)	456(57.1%)	
Lost any ART bottles in past 18 months				
No	1556(98.0%)	773(98.0%)	783(98.0%)	0.9731
Yes	32(2.0%)	16(2.0%)	16(2.0%)	
Location of ART storage				
Home	1259(79.3%)	636(80.6%)	628(78.0%)	
“I keep it with me”	41(2.6%)	15(1.9%)	26(3.3%)	0.3518
Keep ART bottles hidden from others	55(3.5%)	27(3.4%)	28(3.5%)	
Other	233(14.6%)	111(14.1%)	122(15.2%)	
Receiving co‐trimoxazole at baseline				
Yes	1180(74.3%)	207(26.2%)	201(25.2%)	
No	408(25.7%)	582(73.8%)	598(74.8%)	
CD4 at enrolment, copies/mL	580(429−752)	574(410−752)	581(446−749)	0.5807
The viral load was less than 50 copies/mL at enrolment	1538(96.9%)	759(96.2%)	779(97.5%)	0.1763
Treatment plan at enrolment				
2 types of NRTIs+1 type of INSTIs	7 (0.4%)	3 (0.4%)	4(0.5%)	
2 types of NRTIs +1 type of NNRTIs	1450 (91.3%)	715 (90.6%)	735(92.0%)	0.6549
2 types of NRTIs + 1 type of PIs	115 (7.3%)	64 (8.1%)	51 (6.4%)	
Two drug plan	16 (1.0%)	7 (0.9%)	9 (1.1%)	
Follow‐up person‐year	1698	845	853	

*Note*: Data are median (IQR), *n* (%), or *n*/*N* (%).

Abbreviations: ART, antiretroviral therapy; INSTI, integrase strand transfer inhibitor; NRTI, nucleoside reverse transcriptase inhibitor; PI, protease inhibitor.

### 3.2 Treatment Retention

Among 789 participants in the 3‐month dispensing group, 749 were retained at 18 months (94.9%). Among 799 in the 6‐month dispensing group, 753 were retained (94.2%). The risk difference (6‐month minus 3‐month) was −0.7% (95% CI: −2.91% to +1.54%). Since the lower bound of the 95% CI (−2.91%) exceeds the pre‐specified non‐inferiority margin of −5%, non‐inferiority of 6‐month dispensing is demonstrated. The PP analysis showed consistent results (3‐month: 97.59%, 6‐month: 97.75%; RD +0.16%, 95% CI: −0.82% to +1.14%).

### 3.3 Viral Suppression

A total of 1588 participants were included in the final analysis (3‐month group: *n* = 789; 6‐month group: *n* = 799). ITT analysis: In the 3‐month group, 21 participants were lost to follow‐up, resulting in 40 total failure cases (failure rate: 5.07%; viral suppression rate: 94.9%). In the 6‐month group, 28 participants were lost to follow‐up, with 46 total failure cases (failure rate: 5.76%; viral suppression rate: 94.2%). Pearson's chi‐square test showed no significant between‐group difference (χ^2^ = 0.3663, *p* = 0.5405). PP analysis: Definite treatment failure occurred in 19 participants (failure rate: 2.4%; viral suppression rate: 97.6%) in the 3‐month group and 18 participants (failure rate: 2.3%; viral suppression rate: 97.8%) in the 6‐month group. Pearson's chi‐square test indicated no significant between‐group difference (χ^2^ = 0.0421, *p* = 0.8375).

Furthermore, Cox proportional hazards regression analysis further confirmed that regardless of whether lost‐to‐follow‐up cases were considered treatment failures, there was no significant difference in the risk of treatment failure (including virological suppression failure) between the two groups (HR 95% CI 0.5667–2.056, *p* = 0.8149; HR 95% CI 0.5700–1.3270, *p* = 0.5155), indicating that the 6‐month medication regimen is comparable to the 3‐month regimen in terms of virological suppression efficacy.

### 3.4 Cost Analysis

This study also included a detailed analysis of outpatient costs incurred by both patient groups upon achieving the primary outcome (Table 2). The findings revealed that participants in the 6‐month dispensing group averaged two fewer annual outpatient visits than those in the 3‐month group (3 vs. 5 visits, respectively). From the healthcare provider's cost perspective, a 27.7% reduction in treatment expenses was observed in the 6‐month group compared to the 3‐month group (¥931.82 vs. ¥1288.85).

**TABLE 2:** Cost‐effectiveness analysis.

	Overall participants(*n* = 1588)	3‐month dispensing (*n* = 789)	6‐monthbrk dispensing (*n* = 799)
Resource utilization per patient who met the primary outcome			
Clinic visits, median (IQR)	4.00 (3.00−5.00)	5.00 (4.00−5.00)	3.00 (3.00−4.00)
Clinic visits, mean (95% CI)	4.06 (4.00−4.12)	4.73 (4.65−4.80)	3.39 (3.32−3.47)
Missed work to attend current ART clinic visit			
No	980(61.7%)	486(61.6%)	494(61.8%)
Yes	608(38.3%)	303(38.4%)	305(38.2%)
Mean monthly earnings, RMB	4000 (3000−5000)	4000 (3000−5000)	4000 (3000−5000)
Mode of transport to ART clinic			
Walking	144(9.1%)	70(8.9%)	74(9.3%)
Bicycling	40(2.5%)	25(3.2%)	15(1.9%)
Bus	876(55.2%)	430(54.5%)	446(55.8%)
Personal or friend's vehicle	384(24.2%)	195(24.7%)	189(23.7%)
Shared bicycles or electric shared bicycles	74(4.7%)	36(4.6%)	38(4.8%)
Other	70(4.3%)	33(5.1%)	37(4.5%)
One‐way travel time to ART clinic, min	40(30−60)	40(25−60)	40(30−60)
One‐way travel time from home to back home, min	110(60−150)	110(60−150)	110(60−150)
One‐way transport cost to ART clinic, RMB	10(6−20)	10(6−20)	10(6−25)
Time spent at ART clinic, min	40(30−60)	40(25−60)	40(30−60)
Mean provider cost per patient by outcome (95% CI), RMB			
Primary outcome achieved	1068.88 (1048.11−1081.64)	1238.85 (1219.02−1258.69)	891.82 (871.22−912.42)
Primary outcome not achieved	1076.11 (1004.97−1147.24)	1277.12 (1182.82−1371.44)	901.31 (825.36−977.27)
Provider cost breakdown for patients who met the primary outcome			
ART medications	9.11 (4.66−13.57)	9.05 (2.78−15.32)	9.18 (2.83−15.52)
Clinic visits (staff and infrastructure costs allocated per visit)	1055.76 (1039.51−1072.01)	1229.80 (1210.72−1248.88)	882.65 (863.03−902.26)
Patient cost of accessing ART (IQR)			
Median annual work value lost (IQR), US$	209.04 (139.36−278.72)	209.04 (174.20−348.40)	174.20 (104.52−209.04)
Patients with travel cost incurred, *n* (%)	1372(91.4%)	683(91.2%)	689(91.5%)
Median annual cost for patients with travel costs incurred (IQR), RMB	40.00 (20.00−100.00)	50.00 (25.00−100.00)	40.00 (18.00−90.00)

Abbreviation: ART, antiretroviral therapy.

^a^Provider costs: clinic visits (staff time, utilities) and fixed ART care (equipment). Excluded: ART procurement (national free policy), laboratory monitoring (identical between groups), infrastructure depreciation, overhead (non‐incremental).

^b^ART medication cost = patient out‐of‐pocket co‐payment only (98.9% received ART free; 1.1% on non‐first‐line regimens paid median ¥9.11).

^c^Productivity loss: Liaoning Province average wage (not minimum wage).

^d^Data: mean (95% CI), *n* (%), or median (IQR).

Furthermore, work productivity loss attributable to clinic visits was reduced by 16.67% in the 6‐month dispensing group (annual losses: ¥174.20 vs. ¥209.04 in the 3‐month group). Across all evaluated cost metrics, the 6‐month dispensing regimen consistently demonstrated superior cost‐effectiveness relative to the 3‐month protocol.

## 4 Discussion

This individually randomized trial demonstrates that 6‐month ART dispensing maintained non‐inferior treatment retention and viral suppression compared to 3‐monthly dispensing over 18 months, while substantially reducing total costs and patient‐borne burdens. To our knowledge, this is the first individually randomized MMD evidence from Asia, addressing a critical gap given that existing randomized controlled trials derive predominantly from sub‐Saharan Africa.

Comparable clinical outcomes likely reflect reduced structural barriers to adherence—fewer clinic visits lowered transportation costs, time expenditure and work absenteeism for this clinically stable population. The marginally higher loss to follow‐up in the 6‐month group (28 [3.5%] vs. 21 [2.7%]) suggests reduced contact frequency may disproportionately affect vulnerable patients, though absolute differences were small. Detailed tracing of reasons (e.g. migration, stigma‐related avoidance, treatment fatigue) was beyond routine data collection, representing a gap for future studies with systematic tracing protocols. Regarding virologic failure, very low rates in both arms (ITT: 5.07% vs. 5.76%; PP: 2.41% vs. 2.25%) indicate 6‐month dispensing did not compromise viral suppression.

Our findings align with and extend prior randomized evidence. In South African adherence clubs, Cassidy et al. demonstrated non‐inferior 24‐month retention with 6‐ versus 2‐month refills [23]. The INTERVAL trial in Malawi and Zambia showed similar non‐inferiority at 12 months against a pre‐specified margin [13]. Our individually randomized design with a 3‐monthly control arm provides a more stringent test of 6‐month dispensing efficacy, yet yielded consistent results [12]. Notably, our viral suppression rates under a stringent threshold exceeded those reported in the South African cluster‐randomized trial [23], likely reflecting China's hospital‐based care model and concentrated epidemic context. Unlike prior trials [13, 14], our integrated cost analysis demonstrates substantial economic advantages critical for resource allocation decisions.

This study has limitations. The single‐centre design and 18‐month follow‐up preclude generalization to primary healthcare settings and longer‐term outcomes. The predominantly male and MSM‐dominant cohort enhances internal validity but limits applicability to women, heterosexual populations and other key populations. Our eligibility criterion of HIV RNA <200 copies/mL is less stringent than the <50 copies/mL used in some international trials; however, 96.9% of participants were fully suppressed (<50 copies/mL) at enrolment, supporting the validity of our findings. The randomized controlled trial design does not capture patient choice, which is central to DSD models. Cost analysis excluded laboratory, infrastructure and overhead costs. Productivity loss was estimated using the provincial average wage; individual variation may exist, especially for informal employment. ART cost in Table 2 reflects patient co‐payment only (98.9% free under national policy). Exclusion of clinically unstable patients aligns with WHO guidance for MMD eligibility, rather than representing a limitation.

Future research should validate these findings in multicentre settings with extended follow‐up, incorporate complete cost analyses and examine underrepresented sub‐populations, including women and patients with shorter ART duration.

In conclusion, 6‐month dispensing among clinically stable PLHIV in China maintained non‐inferior retention and viral suppression while substantially reducing costs. These findings support WHO recommendations for MMD as a DSD strategy, though they should not be extrapolated to unstable patients or non‐HIV chronic diseases without further evidence.

## Author Contributions

WS, YZ and YH designed the study and wrote the original manuscript under the senior guidance of HS and HD. YZ and YH performed data abstraction. WS, YZ and YH performed data analysis and interpreted the data with support from QH, WG, HS and HD. WS and YZ wrote the first manuscript draft, and YH, QH, XL and WG provided extensive edits to all manuscript drafts. XL, WG and HS contributed to data interpretation and review of all manuscript versions. QH, HSu and HD contributed to data extraction, data quality assessment and data cleaning, as well as providing critical scientific input on the manuscript. All authors approved the final version of the manuscript for publication.

## Funding

The study was funded by the National Science and Technology Major Special Project for Prevention and Control of New Emergencies and Major Infectious Diseases (2025ZD01905001), National Key Research and Development Program Project “Establishment and Optimization of Immunotherapy Protocol for HIV Reservoir Clearance” (2021YFC2301905), Department of Science and Technology of Liaoning Province Project for the High‐Quality Scientific and Technological Development of China Medical University (2023JH2/20200001), Science and Technology Innovation Team of China Medical University (CXTD2022003), and the Non‐profit Central Research Institute Fund of Chinese Academy of Medical Sciences (2023‐PT32001).

## Conflicts of Interest

All authors declare no conflicts of interest. The funder had no role in study design, data collection and analysis, data interpretation, preparation of the manuscript and decision to submit the manuscript for publication.

## Data Availability

Data are available on request from the authors.
